# Improving Distribution Prediction by Integrating Expert Range Maps and Opportunistic Occurrences: Evidence From Japanese Sea Cucumber

**DOI:** 10.1002/ece3.71747

**Published:** 2025-07-06

**Authors:** Bingqing Xiao, Songxi Yuan, Ákos Bede‐Fazekas, Jinxin Zhou, Xingyu Song, Qiang Lin, Lei Cui, Zhixin Zhang

**Affiliations:** ^1^ State Key Laboratory of Tropical Oceanography, Guangdong Provincial Key Laboratory of Applied Marine Biology South China Sea Institute of Oceanology, Chinese Academy of Sciences Guangzhou China; ^2^ University of Chinese Academy of Sciences Beijing People's Republic of China; ^3^ HUN‐REN Centre for Ecological Research Institute of Ecology and Botany Vácrátót Hungary; ^4^ ELTE Eötvös Loránd University, Institute of Geography and Earth Sciences Department of Environmental and Landscape Geography Budapest Hungary; ^5^ Institute of Industrial Science The University of Tokyo Chiba Japan; ^6^ Nansha Marine Ecological and Environmental Research Station Chinese Academy of Sciences Sansha China; ^7^ Key Laboratory of Eutrophication and Red Tide Prevention of Guangdong Higher Education Institutes, College of Life Science and Technology Jinan University Guangzhou China

**Keywords:** data integration, expert range map, opportunistic occurrence, species distribution model, stacked generalization

## Abstract

In an era of biodiversity crisis, it is critical to perform biodiversity assessments to better inform conservation strategies. In this regard, species distribution models (SDMs) represent a widely used tool for biodiversity assessment. Despite their popularity, the accuracy of SDM predictions has long been criticized because we have incomplete or biased information on species distribution. To overcome this limitation, researchers have proposed improving predictions of SDMs by integrating different types of distribution data, but this idea has rarely been explored in the marine realm. In this study, we explored the idea of data integration using the Japanese sea cucumber, whose distribution is known to be restricted by freshwater discharge of the Yangtze River. We first fitted SDMs for this species based on opportunistic occurrence records via four modeling algorithms, then built two types of ensemble models using stacked generalization: an ensemble model that solely used four model predictions and an expert‐informed ensemble model that further accounted for distance to the IUCN expert range map. Our results showed that integrating an expert range map into the opportunistic occurrence model improved distribution prediction by avoiding overprediction in the south of the dispersal barrier for this species. Our study highlights the benefits of integrating expert range maps into opportunistic occurrence SDMs, which improve the reliability of species' spatial distributions.

## Graphical abstract

We first fitted SDMs for the Japanese sea cucumber based on opportunistic occurrence records via four modeling algorithms and then built two types of ensemble models using stacked generalization: an ensemble model that solely used four model predictions, and an expert‐informed ensemble model that further accounted for distance to the IUCN expert range map. Our results showed that integrating an expert range map into the opportunistic occurrence model enhanced model predictive performance and improved range estimates by avoiding overprediction in the south of the dispersal barrier for this species. Our study highlights the benefits of integrating expert range maps into opportunistic occurrence SDMs, which improve the reliability of species' spatial distributions.

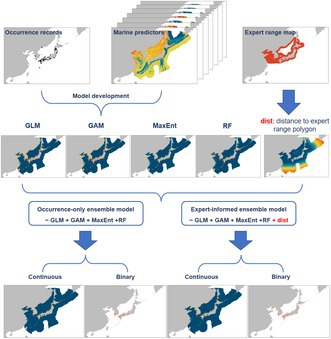

## 1 Introduction

Driven by a combination of threats, such as climate change, habitat degradation, pollution, and biological invasions, biodiversity across the globe is declining more rapidly than ever before (Cowie et al. 2022; Jaureguiberry et al. 2022). In order to maximize conservation efforts and halt the decline of biodiversity, it is of great importance to estimate species' potential distributions accurately. In this regard, species distribution models (SDMs) represent a powerful tool for predicting potential distribution patterns of target species by linking their occurrence records and environmental variables and extrapolating the found correlations through space and time (Araújo et al. 2019; Elith and Leathwick 2009; Guisan et al. 2017). Up to now, researchers have successfully applied SDMs in a number of fields, such as conservation biology (e.g., Franklin 2013; Rodríguez et al. 2007; Velazco et al. 2020), global change biology (e.g., Hu et al. 2021; Qu et al. 2023; Zhang, Ma, et al. 2024), paleoecology (e.g., Wang et al. 2021), and biological invasions (e.g., Zhang, Capinha, et al. 2020; Zhang, Mammola, et al. 2020). Given the vital role of SDMs in biodiversity assessments, the applications of SDMs have increased exponentially during the past few decades (e.g., Araújo et al. 2019; Melo‐Merino et al. 2020; Robinson et al. 2011), and researchers have made considerable efforts to improve model performance, reproducibility, and transparency (e.g., Araújo et al. 2019; Feng et al. 2019; Norberg et al. 2019; Zurell et al. 2020).

Researchers typically use two kinds of distribution data when developing SDMs: opportunistic occurrence records (e.g., Kass et al. 2020; Wang et al. 2021; Qu et al. 2023) and expert range maps (e.g., Boavida‐Portugal et al. 2018; Thuiller et al. 2019; Peng et al. 2023). For instance, Qu et al. (2023) constructed SDMs for seven marine fish species using opportunistic occurrence records, and Thuiller et al. (2019) developed SDMs for ~11,500 species by sampling occurrence records from expert range maps. Despite their popularity in the SDM literature, these two types of distribution data have inherent advantages and drawbacks: opportunistic occurrence records precisely describe species distributions at fine spatial scales but show pronounced sampling biases (Hughes et al. 2021) and lack critical information on dispersal barriers or range limits (Oeser et al. 2024); expert range maps delineate species range limits at coarse spatial resolutions on the basis of expert knowledge (Hurlbert and Jetz 2007) but include substantial unsuitable areas (known as commission error) (Mainali et al. 2020; Merow et al. 2022). Given these remarkable disparities, SDMs based on the two types of distribution data produce different predictions, which confuse conservation practices. For instance, Zhang et al. (2025) constructed SDMs for 233 marine fish species using opportunistic occurrence records and expert range maps, respectively, and found that the similarity levels in habitat suitability predictions between the two models were low. To improve the reliability of SDM predictions and better inform conservation practices, researchers have proposed maximizing the complementary information of different types of distribution data when modeling species distributions, which not only accurately capture the species‐environment associations by using opportunistic occurrences but also reasonably characterize species' range limits with the help of expert range maps (Domisch et al. 2016; Isaac et al. 2020; Merow et al. 2017; Oeser et al. 2024). This emerging field has advanced recently but is still in its infancy; in particular, integrating opportunistic occurrences and expert range maps is extremely rare in the marine realm. The ocean indeed represents an ideal system to test the possibility of data integration because there are a number of dispersal barriers in the ocean, which greatly constrain the geographical distributions of marine organisms (Toonen et al. 2016; Luiz et al. 2012).

In this regard, the Japanese sea cucumber *Apostichopus japonicus* provides an opportunity to explore data integration in the ocean. As an important economic marine species widely distributed along the coastal waters of China, Japan, and Korea, the Japanese sea cucumber has received great research attention, and information on its distribution, both opportunistic occurrence records and expert range map, is abundant (Yang et al. 2015; Zhang, Ma, et al. 2024; Zhang, Zhou, et al. 2024). It has been well recognized that the spatial distribution of the Japanese sea cucumber is geographically constrained by the considerable freshwater outflow of the Yangtze River, which greatly influences marine environmental conditions in the East China Sea (such as salinity and temperature) and limits the southward dispersal of Japanese sea cucumber larvae (Zhang, Ma, et al. 2024; Zhang, Zhou, et al. 2024). However, correlative and physiologically informed SDMs for the Japanese sea cucumber based on opportunistic occurrence records overpredicted the distribution of this species in the south of this biogeographic barrier (e.g., Zhang, Ma, et al. 2024; Zhang, Zhou, et al. 2024). Integration of expert range maps and opportunistic occurrences provides an avenue to improve the realized distribution predictions for this species. In this study, using the Japanese sea cucumber as a case study, we investigated whether integrating an expert range map into an opportunistic occurrence model can improve SDM predictions for this species.

## 2 Materials and Methods

### 2.1 Distribution Data and Marine Predictors

We retrieved opportunistic occurrence records of the Japanese sea cucumber from Zhang, Ma, et al. (2024); Zhang, Zhou, et al. (2024), who collected a total of 458 distribution records from biodiversity databases and scientific literature (Figure S1a). To guarantee the accuracy of occurrence records and avoid clustered records, we excluded records on land and only retained 0.05° (corresponding to approximately 5.5 km at the equator), which corresponds to the spatial resolution of environmental layers. This approach can reduce the effect of sampling bias on models and has been widely used in SDM studies (e.g., Kramer‐Schadt et al. 2013). After data filtering, we kept 222 occurrence records for model development (Figure S1b). We further downloaded the expert range map of the Japanese sea cucumber from the IUCN Red List of Threatened Species (https://www.iucnredlist.org, accessed on July 25, 2024) (Figure S2). 87.8% (195 out of 222) of occurrence records are located within this range.

With respect to marine predictors, considering the fact that the Japanese sea cucumber is a coastal benthic species, we restricted our analyses within the Marine Ecoregions of the World (Spalding et al. 2007) and obtained marine benthic predictors from the Bio‐ORACLE version 3 database (https://www.bio‐oracle.org) (Assis et al. 2024). Laboratory experiments show that temperature (e.g., An et al. 2007), salinity (e.g., Hu et al. 2010), current velocity (e.g., Pan et al. 2015), dissolved oxygen (e.g., Huo et al. 2024), and pH (e.g., González‐Durán et al. 2021) can influence the physiological performance of sea cucumbers. Therefore, we retrieved temperature, salinity, current velocity, dissolved oxygen, and pH‐related predictors (annual mean, maximum, and minimum values) from the Bio‐ORACLE database. Additionally, Zhang, Ma, et al. (2024); Zhang, Zhou, et al. (2024) showed that depth and distance to shore played important roles in determining the spatial distributions of the Japanese sea cucumber; therefore, we further obtained a bathymetry layer from the Bio‐ORACLE database and generated a distance to shore layer. It has been demonstrated that highly correlated predictors can increase the uncertainty of extrapolation (Dormann et al. 2013), and to avoid this issue, we quantified the correlation level between predictors by calculating the Pearson correlation coefficient and selected one predictor among highly correlated predictors (i.e., *r* > 0.7) based on existing knowledge on the ecology of the species. We ultimately considered seven predictors in our analyses, including minimum dissolved oxygen, minimum salinity, annual mean current velocity, minimum current velocity, maximum temperature, water depth, and distance to shore (Figure S3). These marine predictors are at a spatial resolution of 3 arcmin.

### 2.2 Opportunistic Occurrence SDMs

We constructed opportunistic occurrence SDMs for the Japanese sea cucumber via four modeling algorithms, namely generalized linear model (GLM), generalized additive model (GAM), maximum entropy (MaxEnt), and random forest (RF). We chose these four algorithms mainly due to their wide application in predicting the distribution of marine species (e.g., Melo‐Merino et al. 2020; Zhang, Capinha, et al. 2020; Hu et al. 2021; Qu et al. 2023). True absence data are generally not available for marine species, including Japanese sea cucumbers. Hence, we delineated the model calibration area by creating 1000‐km buffers around the occurrence records and selected 10,000 random points within this calibration area as pseudo‐absence data (known as background data for MaxEnt). Empirical evidence supports that a large number of background data is necessary for GLM, GAM, MaxEnt, and RF (Elith and Leathwick 2009; Barbet‐Massin et al. 2012; Valavi et al. 2021). With respect to RF, Valavi et al. (2021) explicitly pointed out that the down‐sampling approach reduces the effect of class imbalance (i.e., imbalance in the number of presence and background data) and shows good predictive performance. Therefore, when fitting the RF model, we followed Valavi et al. (2021) and adopted a down‐sampling approach to handle class imbalance.

Model complexity can greatly affect model performance (e.g., Brun et al. 2020); therefore, best‐practice standards for SDMs recommend optimizing model parameters (Araújo et al. 2019; Feng et al. 2019). Following these guidelines, we optimized model complexity for each algorithm. In short, we fitted GLM with second‐order polynomials via the *glm()* function in the “stats” R package and selected the best model based on the minimum Akaike Information Criterion (Valavi et al. 2022); we fitted GAM with thin plate regression spline smooth term of 3 knots via the *gam()* function in the “mgcv” R package (Wood 2001); we optimized feature class and regularization multiplier for MaxEnt via the *ENMevaluate()* function in the “ENMeval” R package (Kass et al. 2021); with respect to RF, we fitted candidate models with different combinations of key parameters (i.e., the number of trees, the number of predictors sampled at each split, and the minimum size of terminal nodes) via the *randomForest()* function in the “randomForest” R package (Breiman et al. 2018). Among the candidate MaxEnt and RF models, we filtered the best 10% of the models based on their omission rates and then selected the optimal one based on the validation values of the area under the receiver operating characteristic curve (AUC) (see details in Kass et al. 2020; Zhang et al. 2021).

For these selected optimal models, we measured their predictive performance via a 10‐fold random cross‐validation approach. In brief, we randomly divided distribution data into 10 folds; 9 folds were used for model training and the remaining fold for model testing. We repeated this procedure until all folds were used for model testing. We acknowledge that researchers have developed a variety of discrimination metrics to measure the predictive performance of SDMs, such as AUC and true skill statistic (TSS) (Allouche et al. 2006). However, many of these metrics rely on presence‐absence data, and they might be misleading when lacking true absence data (Leroy et al. 2018; Lobo et al. 2008). Among the available discrimination metrics, the continuous Boyce index is considered a reliable measurement for presence‐background SDMs (Hirzel et al. 2006; Leroy et al. 2018). Therefore, we reported the continuous Boyce index in the main text and also presented results about the widely used AUC and TSS in Figure S4. We reported the detailed modeling procedures following the ODMAP protocol in Table S1.

### 2.3 Stacked Generalization

**FIGURE 1:**
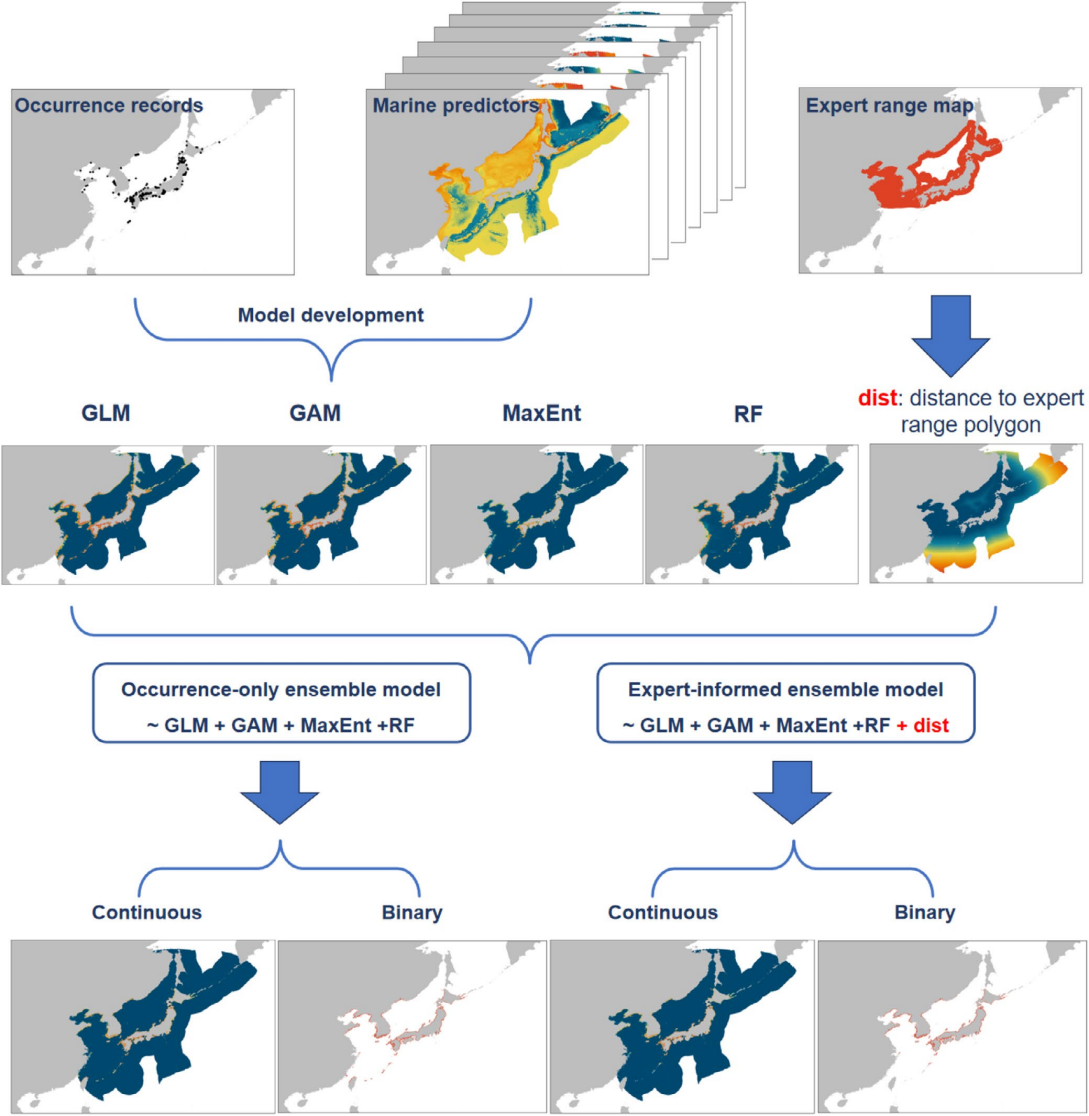
Study design for this study.

Thus far, researchers have developed different strategies to integrate expert range maps into opportunistic occurrence‐based SDMs (e.g., Domisch et al. 2016; Merow et al. 2017; Oeser et al. 2024). For instance, Domisch et al. (2016) incorporated expert range maps by generating a continuous distance‐decay layer, calculated as the distance from each location to the nearest expert‐defined range boundary along the river network. This layer was then used as a covariate in a hierarchical Bayesian SDM. Here, we integrate expert range maps into SDMs using a two‐tier ensemble modeling framework called stacked generalization or stacking (Wolpert 1992) that is widely applied in machine learning (Sesmero et al. 2015) but rarely in species distribution modeling (Bonannella et al. 2022; El Alaoui and Idri 2023; Oeser et al. 2024). First, several simple SDMs (GLM, GAM, MaxEnt, and RF in our research) generate independent probability predictions which are then stacked to form the inputs for the second step: the generalization of the individual predictions (Figure 1; Wolpert 1992; Naimi and Balzer 2018). In this second step, a simple model called meta‐learner takes these inputs as predictors to produce the final occurrence probability predictions (Naimi and Balzer 2018). Practically, the meta‐learner is a binomial GLM (logistic regression). Recently, Oeser et al. (2024) proposed adding one more predictor to the meta‐learner: each pixel's shortest distance to the expert‐defined range polygon. This enables the utilization of a stacked generalization approach for the integration of expert range information with multiple SDM algorithms trained on opportunistic occurrence data.

In brief, we built two types of ensemble models using stacked generalization, that is, with and without distance to expert range polygon. In the first ensemble model (hereafter occurrence‐only ensemble model), we used habitat suitability predictions of these four SDM algorithms as predictors in a logistic regression meta‐learner. In the second ensemble model (hereafter expert‐informed ensemble model), apart from habitat suitability predictions by the four SDM algorithms, we further included distance to the expert range polygon as a predictor in the logistic regression meta‐learner. For each species, we computed a distance layer to the IUCN expert range map, where the distance is 0 if the location is within the IUCN expert range map and increases as the location moves farther outside the range.

To compare the predictive performance of the two ensemble models, we calculated the Boyce values for each ensemble model using a 10‐fold random cross‐validation approach. Additionally, we used the Wilcoxon paired rank sum test to assess whether there were significant differences in predictive performance between the two types of ensemble models. We binarized continuous habitat suitability maps predicted by the two types of ensemble models via a 10% omission rate threshold.

## 3 Results

### 3.1 Predictive Performance

**FIGURE 2:**
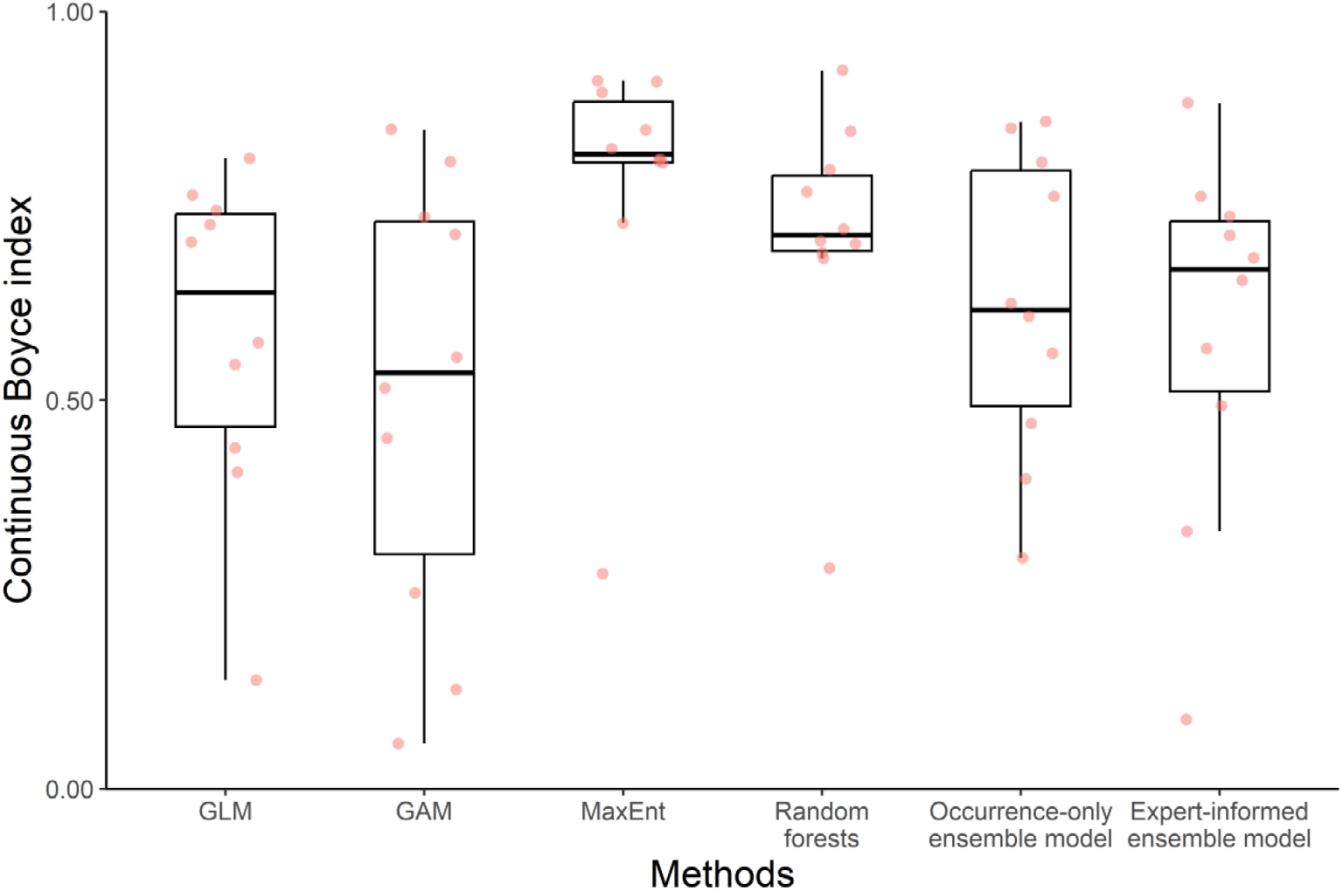
Predictive performance of six species distribution models fitted by different strategies. GAM, generalized additive model; GLM, generalized linear model; MaxEnt, maximum entropy.

Cross‐validation results showed that these six modeling algorithms exhibited good predictive performance, with an average (± standard deviation) Boyce index of 0.633 ± 0.224 (GLM: 0.585 ± 0.199; GAM: 0.506 ± 0.268; MaxEnt: 0.781 ± 0.177; RF: 0.712 ± 0.161; occurrence‐only ensemble model: 0.623 ± 0.185; expert‐informed ensemble model: 0.591 ± 0.222) (Figure 2). We found that among these six methods, machine learning algorithms showed the highest predictive performance (Figure 2). There was no significant difference in the continuous Boyce index values between the expert‐informed and occurrence‐only ensemble models (two‐sided paired Wilcoxon signed rank tests, *V* = 33, *p* = 0.625). The paired Wilcoxon signed‐rank tests revealed only minimal differences in AUC and TSS values across the six models, with no statistically significant differences detected (Figure S4).

**FIGURE 3:**
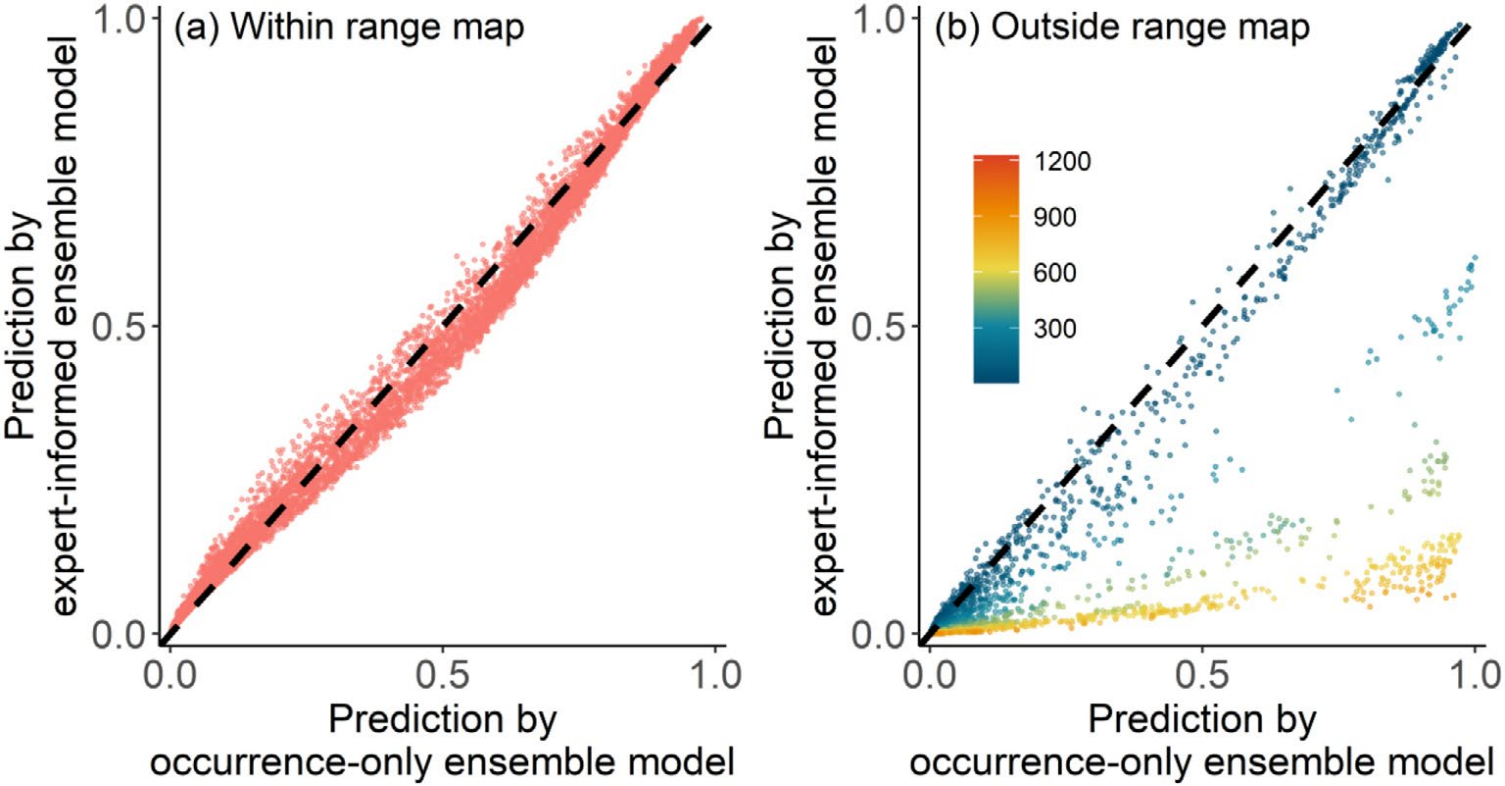
Comparison of habitat suitability predicted by the two types of ensemble models. Habitat suitability predicted by the two ensemble models (a) within and (b) outside expert range map. The dashed lines in panels (a) and (b) show 1:1 reference lines. The color legend in panel (b) indicates the distance to expert range polygon.

In the two types of ensemble models, predictions of GLM and GAM did not significantly influence the results, whereas predictions of MaxEnt and RF were significantly associated with the results (Table 1). The distance to the expert range was identified as a significant predictor variable in the expert‐informed ensemble model (Table 1). In the expert‐informed ensemble model, the distance layer had relatively minor effects on habitat suitability predictions within the expert range polygon and was negatively associated with species habitat suitability outside the expert range polygon (Figure 3; Figures S5 and S6).

**TABLE 1:** Coefficients and associated *p* values of two ensemble models fitted by logistic regression meta‐learner.

Predictor variable	Occurrence‐only ensemble model		Expert‐informed ensemble model	
	Coefficient	*p*	Coefficient	*p*
GLM	1.835	0.652	0.423	0.907
GAM	−3.878	0.361	−2.356	0.531
MaxEnt	1.887	0.002	2.560	< 0.001
RF	9.027	< 0.001	7.528	< 0.001
Distance to expert range	—	—	−0.004	< 0.001

### 3.2 Model Predictions

**FIGURE 4:**
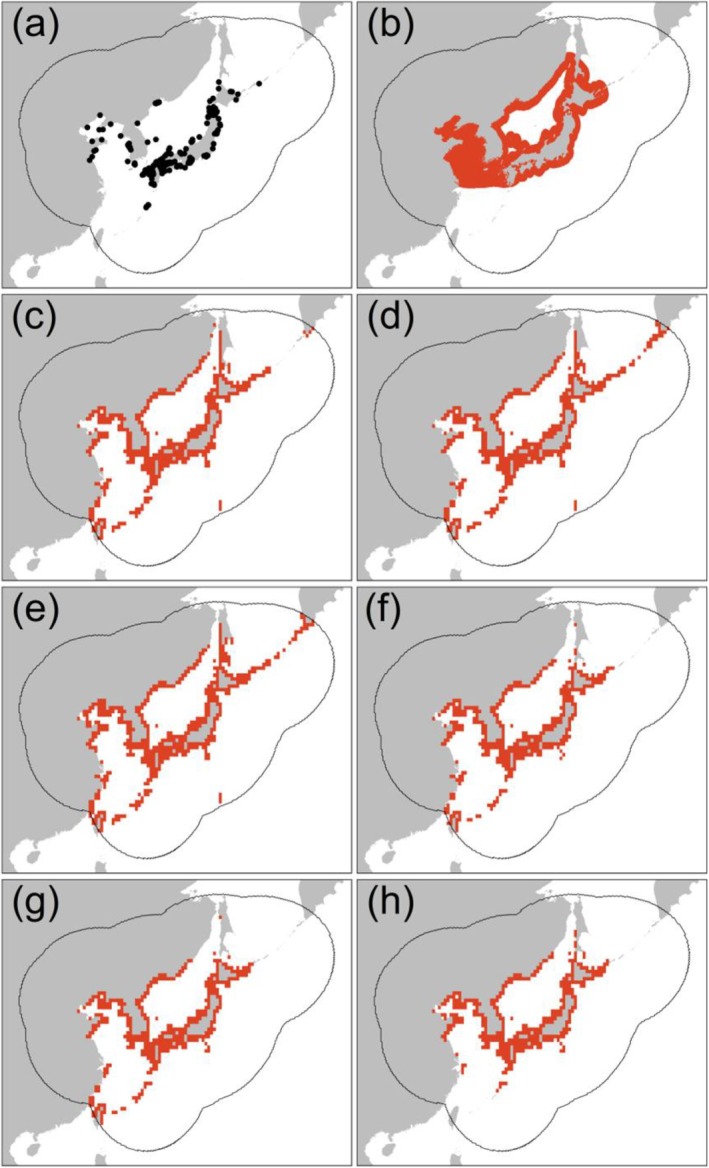
Spatial distribution patterns of the Japanese sea cucumber predicted by species distribution models. (a) Opportunistic occurrence records, (b) IUCN expert range map, (c) spatial prediction by generalized linear model, (d) spatial prediction by generalized additive model, (e) spatial prediction by maximum entropy, (f) spatial prediction by random forest, (g) spatial prediction by occurrence‐only ensemble model, and (h) spatial prediction by expert‐informed ensemble model. To improve visualization of maps, we aggregated habitat suitability maps into 30 arcmin spatial resolution. High‐resolution figures can be found in Figure S6.

The four modeling algorithms predicted different suitable range sizes for the Japanese sea cucumber, ranging from 125,213 km^2^ (RF) to 183,826 (MaxEnt), but they consistently predicted suitable ranges in the south of the Yangtze River Estuary, where this species does not naturally occur (Figure 4; Figures S7 and S8). With respect to the two types of ensemble models, they predicted largely different spatial distributions in several aspects. First, the expert‐informed ensemble model (119,810 km^2^) predicted a smaller suitable range size than the occurrence‐only ensemble model (121,965 km^2^). Second, compared with the occurrence‐only ensemble model, the expert‐informed ensemble model successfully predicted the absence of the Japanese sea cucumber in the south of the Yangtze River Estuary (Figure 4; Figures S7 and S8). The southernmost latitude of the suitable range predicted by the occurrence‐only ensemble model was 22.975°N, whereas the expert‐informed ensemble model estimated it at 27.325°N. The latter aligns more closely with the species' known southern range limit (30.775° N according to the IUCN expert range map). Third, the expert‐informed ensemble model (92.4%) predicted a higher proportion of suitable ranges within the IUCN expert range map than that of the occurrence‐only ensemble model (86.9%). Our above findings together support that integrating expert range maps into opportunistic occurrence SDMs can improve species distribution prediction.

## 4 Discussion

In this study, we highlight the benefit of integrating expert range maps in opportunistic occurrence SDMs using the Japanese sea cucumber, whose distribution is known to be constrained by an oceanic dispersal barrier. Our results clearly show that the expert‐informed ensemble model that accounts for the expert range map can improve the reliability of SDM predictions by avoiding overprediction beyond dispersal limitation. Based on our findings, we encourage researchers to incorporate expert knowledge when predicting species spatial distributions, which will improve prediction reliability and better inform marine biodiversity conservation, especially in an era of climate and biodiversity crises.

### 4.1 The Importance of Data Integration

Researchers have long recognized that species spatial distributions are determined by multiple factors, including biotic interactions, abiotic conditions, and mobility, which is widely known as the BAM framework (Soberón and Peterson 2005). Besides, researchers recently acknowledged the importance of additional sources of knowledge, such as physiological tolerance (Feng et al. 2020; Zhang, Ma, et al. 2024; Zhang, Zhou, et al. 2024), intraspecific variation (Fitzpatrick and Keller 2015; Zhang et al. 2021), and human influence (Feng et al. 2024; Frans and Liu 2024), on species distributions. Therefore, there is a clear need to account for multiple sources of information in SDMs beyond abiotic predictors to improve species' distribution prediction. Apart from the above‐mentioned multiple sources of information, range limits constrained by factors like biotic interactions and dispersal barriers are important but largely neglected information influencing species' distributional ranges (Oeser et al. 2024), and SDMs ignoring range limits are at risk of overpredicting species distributions. Informed by expert knowledge, expert range maps provide information on the range limits of target species by capturing dispersal barriers and biological constraints. For instance, in our case, the distribution of the Japanese sea cucumber is constrained by the freshwater discharge of the Yangtze River; thus, larvae of this species are unable to spread south of the Yangtze Estuary (Yang et al. 2015). However, previous SDMs based on opportunistic occurrences overpredicted the distribution of this species in the south of the Yangtze Estuary (Zhang, Ma, et al. 2024; Zhang, Zhou, et al. 2024), which clearly calls for integrating range limit information into SDMs to improve species' distribution prediction. In this study, using the data‐driven approach proposed by Oeser et al. (2024), we accounted for information on the range extent of the Japanese sea cucumber provided by the IUCN expert range map in opportunistic occurrence SDMs. Our findings clearly showed that the expert‐informed ensemble model improved the reliability of species distributions by successfully predicting the absence of the Japanese sea cucumber in the south of the Yangtze Estuary. Given the effective data integration approach (e.g., Domisch et al. 2016; Merow et al. 2017; Oeser et al. 2024), promising results of our study, and the readily available information on species' range limits in the IUCN database, we recommend researchers improve distribution prediction by integrating the complementary information provided by the two types of distribution data.

Expert range maps and opportunistic occurrence records have been widely used in conservation, ecology, macroecological, and biogeographical studies, yet they produced confusing results (Alhajeri and Fourcade 2019; Aronsson et al. 2024; Fourcade 2016; Herkt et al. 2017; Hurlbert and White 2005; Zhang et al. 2025). For instance, Alhajeri and Fourcade (2019) focused on 1315 rodent species and reported that environmental data estimates derived from expert range maps and opportunistic occurrence records were highly correlated, suggesting both types of distribution data can be used for macroecological inferences. However, Zhang et al. (2025) constructed SDMs for 233 marine fish species using expert range maps and opportunistic occurrence records independently and found substantial differences in distribution prediction between the two types of distribution models. We presently have incomplete or biased knowledge of species distribution (e.g., Alhajeri and Fourcade 2019; Herkt et al. 2017; Hughes et al. 2021); besides, both expert range maps and opportunistic occurrence records contain important information on species distributions. Therefore, instead of arguing the superiority of one distribution data type over the other, we strongly advocate for integrating the complementary information provided by different types of distribution data. Meanwhile, we caution that the effects of data integration on species' distribution prediction largely depend on the accuracy of expert range maps, which was also explicitly highlighted by Oeser et al. (2024). In our case, the Japanese sea cucumber has been well studied and an expert range map from the IUCN properly delineates its range limits; consequently, our expert‐informed ensemble model yielded satisfying distribution prediction for this species. Given the fact that our understanding of species distributions may be biased and expert range maps for marine species might be incomplete, the effectiveness of data integration might be species specific and needs to be examined carefully.

### 4.2 Be Cautious About Performance Metrics

Our results showed that the expert‐informed ensemble model produced more reliable distribution predictions for the Japanese sea cucumber compared to that of the occurrence‐only ensemble model. The superiority of the expert‐informed ensemble model over the occurrence‐only ensemble model was not supported by the continuous Boyce index. Our results showed that tuned machine learning algorithms outperformed ensemble models; a similar finding was previously reported by Hao et al. (2020). These results suggest that during modeling, we should not make a decision (e.g., modeling algorithm selection and predictor set selection) by relying too much on these discrimination metrics. Similar warning messages have been clearly expressed in the literature (e.g., Bede‐Fazekas and Somodi 2020; Huang et al. 2025; Santini et al. 2021; Zhang, Ma, et al. 2024; Zhang, Zhou, et al. 2024). For instance, Santini et al. (2021) used a virtual species approach and explicitly pointed out that researchers should not justify model reliability based on discrimination metrics. This is especially true for distribution models fitted with presence‐only or presence‐background data (Leroy et al. 2018). As a result, researchers should pay due attention to the choice of discrimination metrics and properly interpret associated results.

## Author Contributions

**Bingqing Xiao:** data curation (lead), formal analysis (equal), methodology (equal), visualization (equal), writing – original draft (equal). **Songxi Yuan:** formal analysis (equal), methodology (equal), visualization (equal), writing – original draft (equal). **Ákos Bede‐Fazekas:** methodology (supporting), writing – review and editing (supporting). **Jinxin Zhou:** data curation (supporting), methodology (supporting), supervision (equal). **Xingyu Song:** writing – original draft (supporting), methodology (supporting). **Qiang Lin:** supervision (equal), resources (lead), validation (equal). **Lei Cui:** methodology (equal), supervision (equal), validation (equal). **Zhixin Zhang:** conceptualization (lead), writing – review and editing (lead), validation (lead).

## Conflicts of Interest

The authors declare no conflicts of interest.

## Supporting information


Data S1.
**Figure S1.** Opportunistic occurrence records of the Japanese sea cucumber. (a) Collected occurrence records, and (b) occurrence records after data cleaning.
**Figure S2.** The expert range map of the Japanese sea cucumber sourced from the IUCN Red List of Threatened Species.
**Figure S3.** Collinearity between 17 marine predictors. cv, current velocity; dshore, distance to shore; max, annual maximum value; mean, annual mean value; min, annual minimum value; o_2_, dissolved molecular oxygen; range, annual range value; sss, sea surface salinity; sst, sea surface temperature.
**Figure S4.** Predictive performance of six species distribution models measured by AUC and TSS. AUC, area under the receiver operating characteristic curve; GAM, generalized additive model; GLM, generalized linear model; MaxEnt, maximum entropy; TSS, true skill statistic.
**Figure S5.** Relationships between distance to expert range polygon and continuous habitat suitability predictions by species distribution models. Habitat suitability prediction by (a) generalized linear model, (b) generalized additive model, (c) maximum entropy, (d) random forest, (e) occurrence‐only ensemble model, and (f) expert‐informed ensemble model. The colors show the number grid cells. To improve visualization, we used the same color as for the value 50,000 for values over 50,000. The red lines are fitted by generalized additive models.
**Figure S6.** Relationships between distance to expert range polygon and binary habitat suitability predictions by species distribution models. Habitat suitability prediction by (a) generalized linear model, (b) generalized additive model, (c) maximum entropy, (d) random forest, (e) occurrence‐only ensemble model, and (f) expert‐informed ensemble model. The colors show the number grid cells. To improve visualization, we used the same color as for the value 50,000 for values over 50,000. The red lines are fitted by generalized additive models.
**Figure S7.** Spatial distribution patterns of the Japanese sea cucumber predicted by species distribution models. Binary habitat suitability predictions by (a) generalized linear model, (b) generalized additive model, (c) maximum entropy, (d) random forest, (e) occurrence‐only ensemble model, and (f) expert‐informed ensemble model. The dashed lines indicate the southernmost latitude of the IUCN expert range map. Red regions indicate suitable ranges predicted by distribution models.
**Figure S8.** Spatial distribution patterns of the Japanese sea cucumber predicted by species distribution models. Continuous habitat suitability predictions by (a) generalized linear model, (b) generalized additive model, (c) maximum entropy, (d) random forest, (e) occurrence‐only ensemble model, and (f) expert‐informed ensemble model.
**Table S1.** ODMAP (overview, data, model, assessment, prediction) metadata for the methodology used in this study.

## Data Availability

Data and R scripts for this study are publicly available in Figshare Repository (https://figshare.com/s/35f35be317e789b97a29).
