# Supplementary material for: Improving Distribution Prediction by Integrating Expert Range Maps and Opportunistic Occurrences: Evidence From Japanese Sea Cucumber
Source: Ecol Evol. 2025 Jul 6;15(7):e71747. doi: 10.1002/ece3.71747 (PMC12229810; doi:10.1002/ece3.71747)
Supplement: Supplementary file 1 — Data S1. Figure S1. Opportunistic occurrence records of the Japanese sea cucumber. (a) Collected occurrence records, and (b) occurrence records after data cleaning. Figure S2. The expert range map of the Japanese sea cucumber sourced from the IUCN Red List of Threatened Species. Figure S3. Collinearity between 17 marine predictors. cv, current velocity; dshore, distance to shore; max, annual maximum value; mean, annual mean value; min, annual minimum value; o2, dissolved molecular oxygen; range, annual range value; sss, sea surface salinity; sst, sea surface temperature. Figure S4. Predictive performance of six species distribution models measured by AUC and TSS. AUC, area under the receiver operating characteristic curve; GAM, generalized additive model; GLM, generalized linear model; MaxEnt, maximum entropy; TSS, true skill statistic. Figure S5. Relationships between distance to expert range polygon and continuous habitat suitability predictions by species distribution models. Habitat suitability prediction by (a) generalized linear model, (b) generalized additive model, (c) maximum entropy, (d) random forest, (e) occurrence‐only ensemble model, and (f) expert‐informed ensemble model. The colors show the number grid cells. To improve visualization, we used the same color as for the value 50,000 for values over 50,000. The red lines are fitted by generalized additive models. Figure S6. Relationships between distance to expert range polygon and binary habitat suitability predictions by species distribution models. Habitat suitability prediction by (a) generalized linear model, (b) generalized additive model, (c) maximum entropy, (d) random forest, (e) occurrence‐only ensemble model, and (f) expert‐informed ensemble model. The colors show the number grid cells. To improve visualization, we used the same color as for the value 50,000 for values over 50,000. The red lines are fitted by generalized additive models. Figure S7. Spatial distribution patterns of the Japanes [file ECE3-15-e71747-s001.docx]

# **Supporting Figures**

**Figure S1.** Opportunistic occurrence records of the Japanese sea cucumber. (a) Collected occurrence records, and (b) occurrence records after data cleaning.


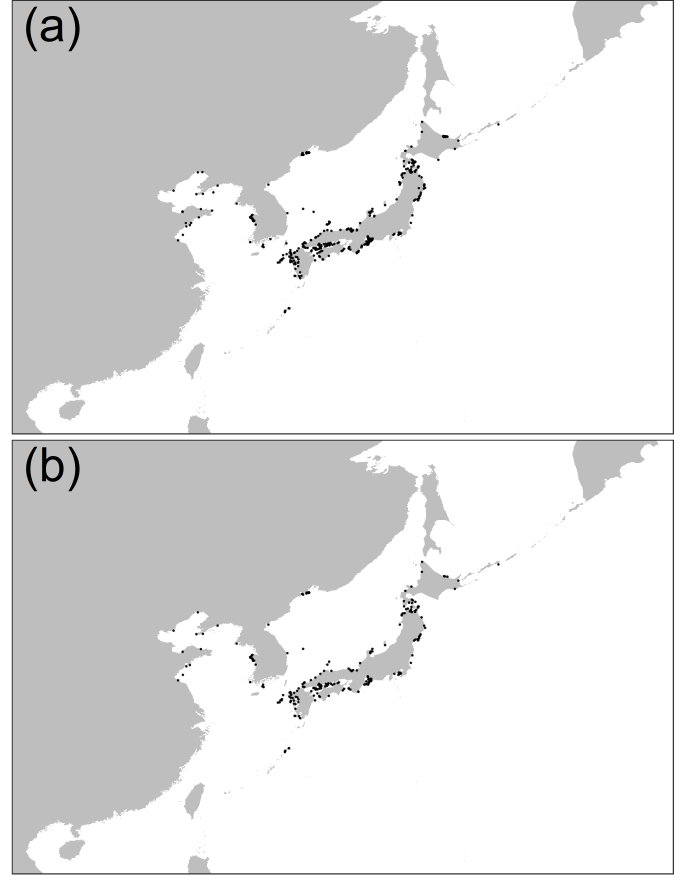


**Figure S2.** The expert range map of the Japanese sea cucumber sourced from the IUCN Red List of Threatened Species.

**
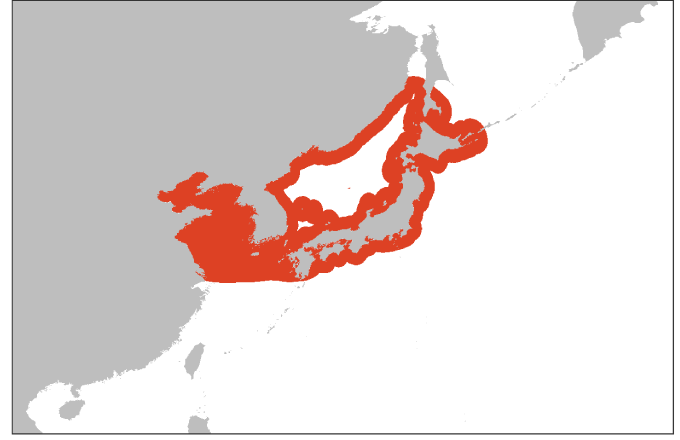
**

**Figure S3.** Collinearity between 17 marine predictors. cv, current velocity; dshore, distance to shore; max, annual maximum value; mean, annual mean value; min, annual minimum value; o2, dissolved molecular oxygen; range, annual range value; sss, sea surface salinity; sst, sea surface temperature.


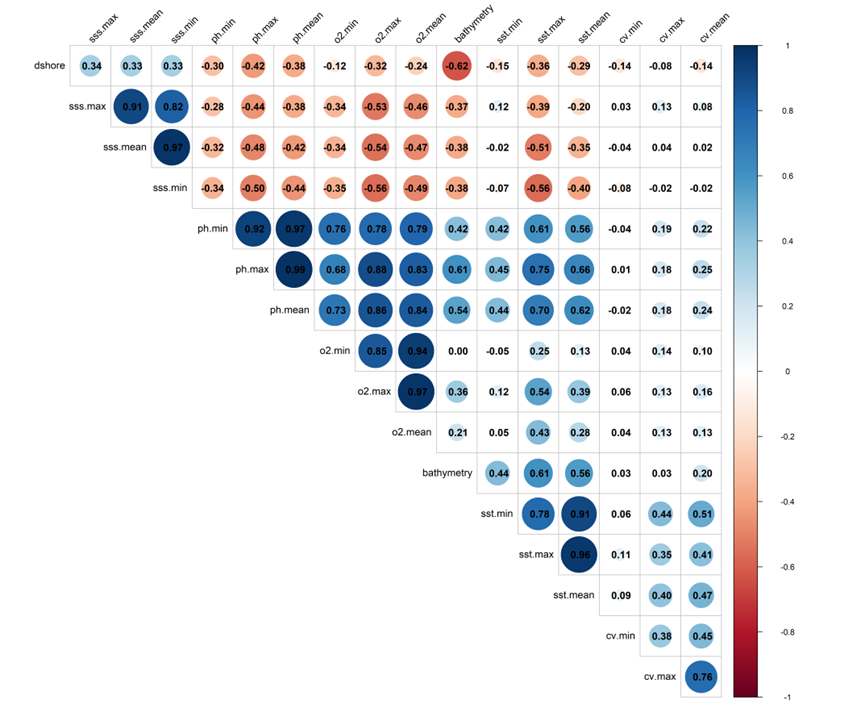


**Figure S4.** Predictive performance of six species distribution models measured by AUC and TSS. AUC, area under the receiver operating characteristic curve; GAM, generalized additive model; GLM, generalized linear model; MaxEnt: maximum entropy; TSS, true skill statistic.


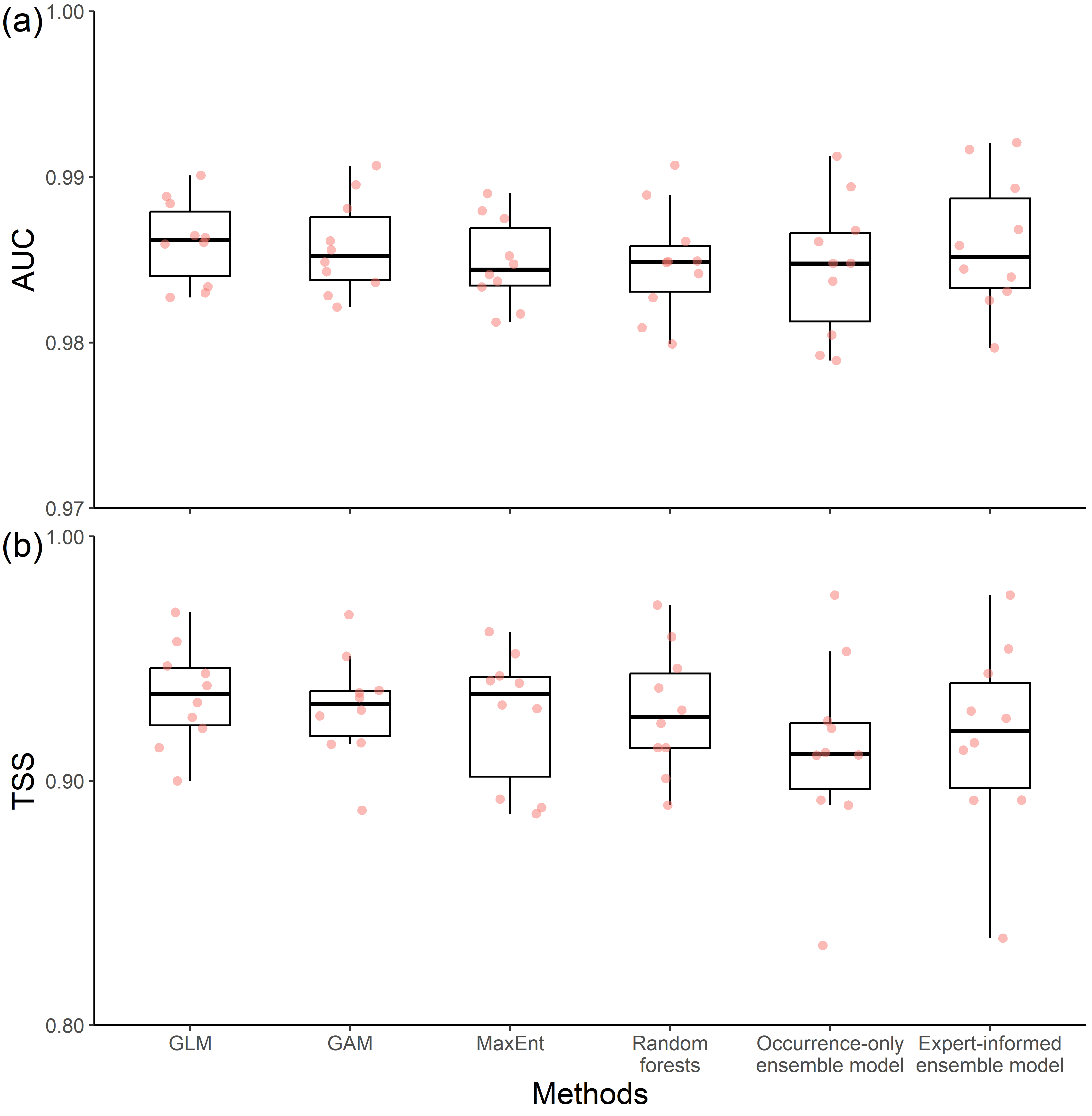


**Figure S5.** Relationships between distance to expert range polygon and continuous habitat suitability predictions by species distribution models. Habitat suitability prediction by (a) generalized linear model, (b) generalized additive model, (c) maximum entropy, (d) random forest, (e) occurrence-only ensemble model, and (f) expert-informed ensemble model. The colors show the number grid cells. To improve visualization, we used the same color as for the value 50,000 for values over 50,000. The red lines are fitted by generalized additive models.

**
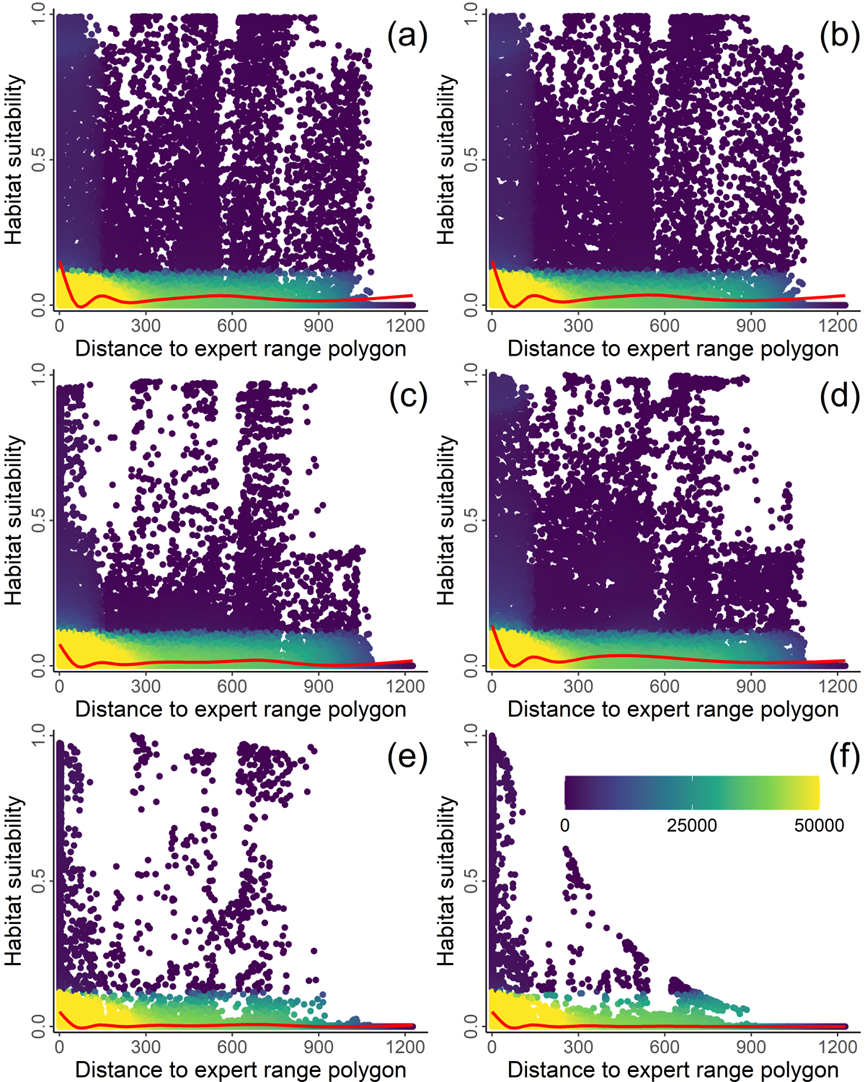
**

**Figure S6.** Relationships between distance to expert range polygon and binary habitat suitability predictions by species distribution models. Habitat suitability prediction by (a) generalized linear model, (b) generalized additive model, (c) maximum entropy, (d) random forest, (e) occurrence-only ensemble model, and (f) expert-informed ensemble model. The colors show the number grid cells. To improve visualization, we used the same color as for the value 50,000 for values over 50,000. The red lines are fitted by generalized additive models.

**
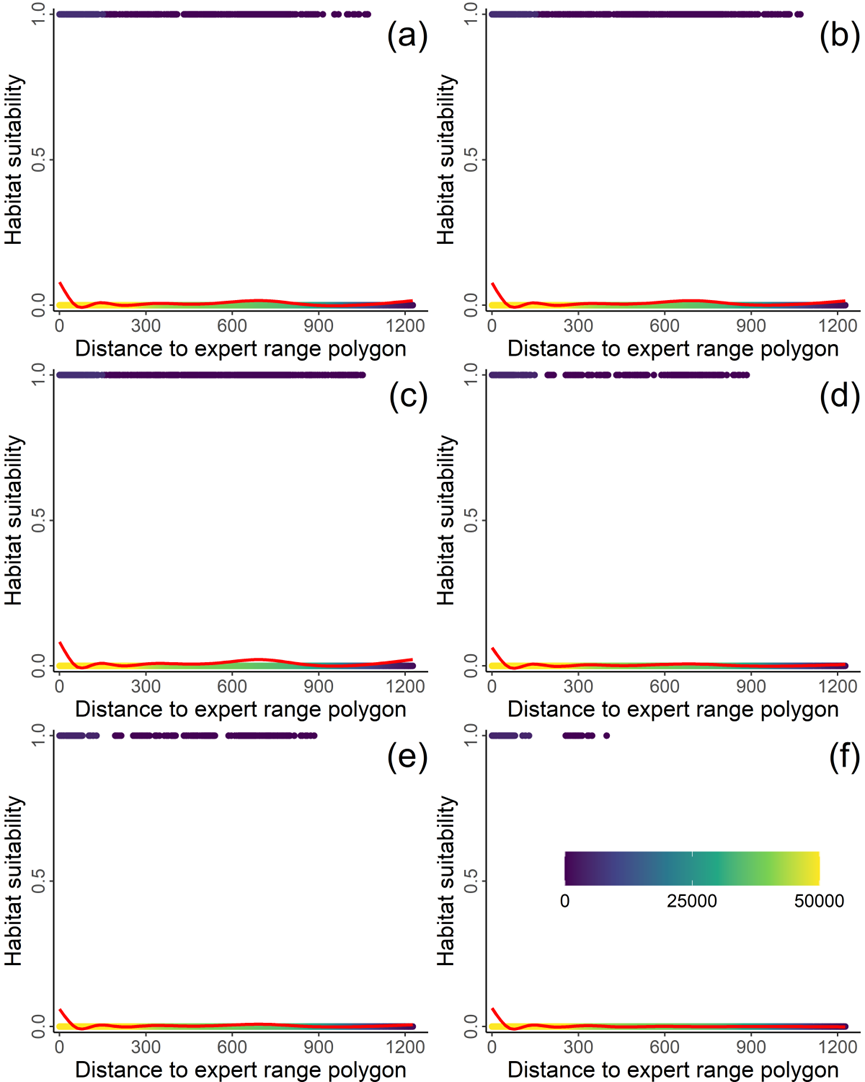
**

**Figure S7.** Spatial distribution patterns of the Japanese sea cucumber predicted by species distribution models. Binary habitat suitability predictions by (a) generalized linear model, (b) generalized additive model, (c) maximum entropy, (d) random forest, (e) occurrence-only ensemble model, and (f) expert-informed ensemble model. The dashed lines indicate the southernmost latitude of the IUCN expert range map. Red regions indicate suitable ranges predicted by distribution models.


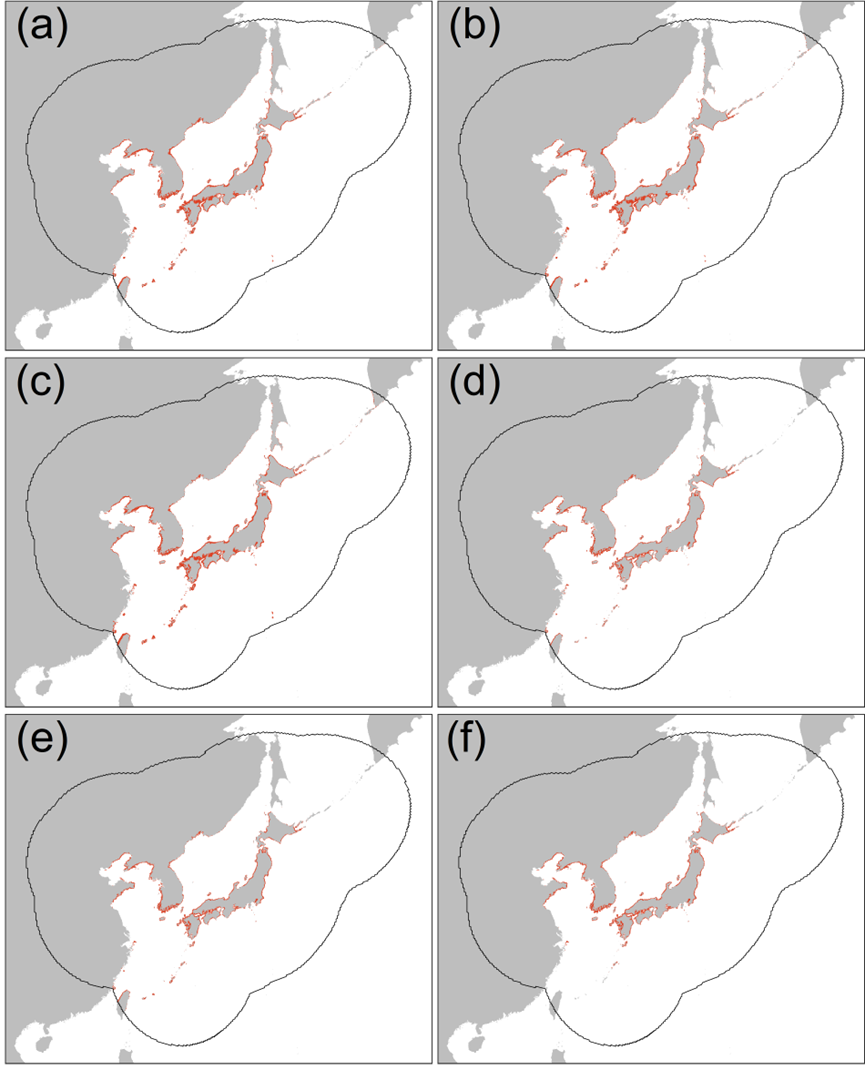


**Figure S8.** Spatial distribution patterns of the Japanese sea cucumber predicted by species distribution models. Continuous habitat suitability predictions by (a) generalized linear model, (b) generalized additive model, (c) maximum entropy, (d) random forest, (e) occurrence-only ensemble model, and (f) expert-informed ensemble model.


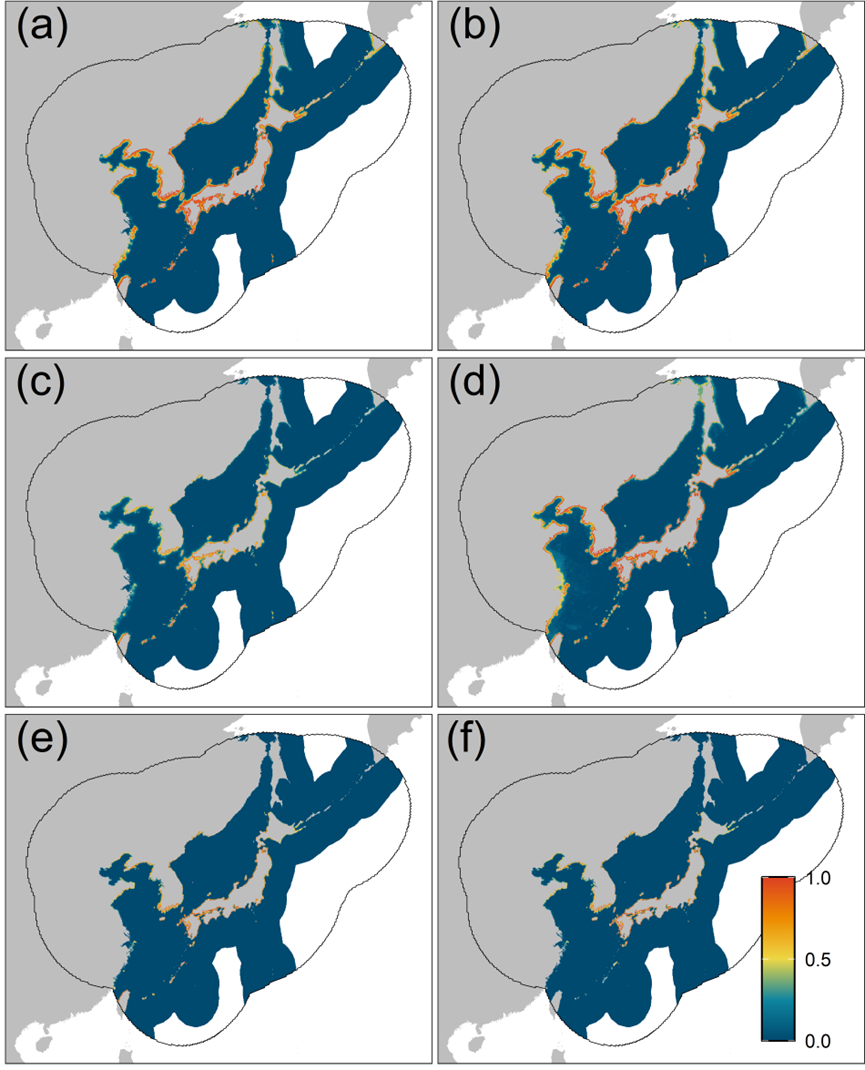


# **Supporting Table**

**Table S1.** ODMAP (overview, data, model, assessment, prediction) metadata for the methodology used in this study.

| Section | Subsection | Element | Value |
| --- | --- | --- | --- |
| Overview | Authorship | Study title | Improving distribution prediction by integrating expert range maps and opportunistic occurrences: evidence from Japanese sea cucumber |
| Overview | Authorship | Author names | Bingqing Xiao; Songxi Yuan; Jinxin Zhou; Ákos Bede-Fazekas; Xingyu Song; Qiang Lin; Zhixin Zhang |
| Overview | Authorship | Contact | zxzhang@scsio.ac.cn |
| Overview | Authorship | Study link | Not yet provided |
| Overview | Model objective | Model objective | Mapping and interpolation |
| Overview | Model objective | Target output | Habitat suitability |
| Overview | Focal Taxon | Focal Taxon | Japanese sea cucumber: *Apostichopus japonicus* |
| Overview | Location | Location | The coastal waters of China, Japan, and Korea |
| Overview | Scale of Analysis | Spatial extent | 107.2903, 161.7986, 18.92523, 54.61063 (xmin, xmax, ymin, ymax) |
| Overview | Scale of Analysis | Spatial resolution | 3 arcmin |
| Overview | Scale of Analysis | Temporal extent | 2010–2020 |
| Overview | Scale of Analysis | Temporal resolution | Not applicable |
| Overview | Scale of Analysis | Boundary | Political |
| Overview | Biodiversity data | Observation type | Field survey, range map |
| Overview | Biodiversity data | Response data type | point occurrence |
| Overview | Predictors | Predictor types | climatic; topographic |
| Overview | Hypotheses | Hypotheses | Mounting evidence highlights that temperature (e.g., An et al. 2007), salinity (e.g., Hu et al. 2010), current velocity (e.g., Pan et al. 2015), dissolved oxygen (e.g., Huo et al. 2024), and pH (e.g., González-Durán, 2021) can influence the physiological performance of sea cucumbers. Moreover, Zhang et al. (2024) showed that depth and distance to shore played important roles in determining the spatial distributions of the Japanese sea cucumber. |
| Overview | Assumptions | Model assumptions | Species-environment equilibrium, Availability of all important predictors |
| Overview | Algorithms | Modelling techniques | Generalized linear model (GLM), generalized additive model (GAM), maximum entropy (MaxEnt), and ranfom forests (RF) |
| Overview | Algorithms | Model complexity | We fitted GLM with second-order polynomials via the glm() function in the “stats” R package and selected the best model based on the minimum Akaike Information Criterion (AIC) (Valavi et al. 2022); we fitted GAM with thin plate regression spline smooth term of 3 knots via the gam() function in the “mgcv” R package (Wood 2001); we optimized feature class and regularization multiplier for MaxEnt via the ENMevaluate() function in the “ENMeval” R package (Kass et al. 2021); with respect to RF, we fitted candidate models with different combinations of key parameters (i.e., the number of trees, the number of predictors sampled at each split, and the minimum size of terminal nodes) via the randomForest() function in the “randomForest” R package (Breiman et al. 2018). Among the candidate MaxEnt and RF models, we filtered the best 10% of the models based on their omission rates, and then selected the optimal one based on the validation AUC values (see details in Kass et al. 2020; Zhang et al. 2021). |
| Overview | Algorithms | Model averaging | We built two types of ensemble models, i.e., with and without distance to expert range polygon maps, using stacked generalization. In the first ensemble model, we used habitat suitability predictions of these four SDM algorithms as predictors in a logistic regression meta-learner. In the second ensemble model, apart from habitat suitability predictions by the four SDM algorithms, we further included distance to the expert range polygon as a predictor in the logistic regression meta-learner. |
| Overview | Workflow | Model workflow | We collected distribution data from Zhang et al. (2024). We reduced sampling bias by keeping one record per 3 arcmin grid cell. We selected marine predictors based on their ecological relevance and collinearity. We fitted SDMs using GLM, GAM, MaxEnt, Randomforest. We assessed model performance using five-fold cross-validation approach. We predicted Japanese sea cucumber distribution under present (2010-2020). |
| Overview | Software | Software | We performed all the analyses in R version 4.3.3, mainly using ENMeval, raster, sf, mgcv, randomForest R packages. |
| Overview | Software | Code availability | R scripts used for this study are available in Figshare Repository (https://figshare.com/s/35f35be317e789b97a29). |
| Overview | Software | Data availability | Species distribution data and marine predictors are available in Figshare Repository (https://figshare.com/s/35f35be317e789b97a29). |
| Data | Biodiversity data | Taxon names | *Apostichopus japonicus* |
| Data | Biodiversity data | Taxonomic reference system | Yang, H., Hamel, J. F., & Mercier, A. (2015). The sea cucumber *Apostichopus japonicus*: History, biology and aquaculture. Springer. |
| Data | Biodiversity data | Ecological level | species |
| Data | Biodiversity data | Data sources | We collected data from Zhang et al. (2024) and download expert range map from IUCN Red List(https://www.iucnredlist.org/). |
| Data | Biodiversity data | Sampling design | NA |
| Data | Biodiversity data | Sample size | NA |
| Data | Biodiversity data | Clipping | NA |
| Data | Biodiversity data | Scaling | No |
| Data | Biodiversity data | Cleaning | To guarantee the accuracy of occurrence records and avoid clustered records, we excluded records on land and only retained a single occurrence per 3-arcmin grid cell, which corresponds to the spatial resolution of environmental layers. |
| Data | Biodiversity data | Absence data | None |
| Data | Biodiversity data | Background data | We created 1,000 km buffer around species occurrence records, then selected 10,000 random points as background data. |
| Data | Biodiversity data | Errors and biases | We reduced sampling bias by keeping one record per 3 armin grid cell. |
| Data | Data partitioning | Training data | five-fold random cross-validation |
| Data | Data partitioning | Validation data | withheld from model fitting |
| Data | Data partitioning | Test data | NA |
| Data | Predictor variables | Predictor variables | We ultimately considered 7 predictors in our analyses, including minimum dissolved oxygen, annual range of dissolved oxygen, minimum pH, minimum salinity, annual mean current velocity, minimum current velocity, maximum temperature, water depth, and distance to shore. |
| Data | Predictor variables | Data sources | Bio-ORACLE version 3 database (https://www.bio-oracle.org). |
| Data | Predictor variables | Spatial extent | 107.2903, 161.7986, 18.92523, 54.61063 (xmin, xmax, ymin, ymax) |
| Data | Predictor variables | Spatial resolution | 3 arcmin |
| Data | Predictor variables | Coordinate reference system | WGS84 (EPSG:4326) |
| Data | Predictor variables | Temporal extent | 2010–2020 |
| Data | Predictor variables | Temporal resolution | NA |
| Data | Predictor variables | Data processing | We quantified the correlation levels between abiotic predictors via the Pearson’s correlation coefficient (*r*) and excluded strongly collinear variables (i.e., \|*r*\| > 0.7) to reduce their possible effects on model fitting (Dormann et al. 2013). |
| Data | Predictor variables | Errors and biases | NA |
| Data | Predictor variables | Dimension reduction | Based on collinearity results, we selected seven non-collinear variables. |
| Data | Transfer data | Data sources | Bio-ORACLE version 3 database (https://www.bio-oracle.org). |
| Data | Transfer data | Spatial extent | 107.2903, 161.7986, 18.92523, 54.61063 (xmin, xmax, ymin, ymax) |
| Data | Transfer data | Spatial resolution | 3 arcmin |
| Data | Transfer data | Temporal extent | 2010–2020 |
| Data | Transfer data | Temporal resolution | NA |
| Data | Transfer data | Models and scenarios | NA |
| Data | Transfer data | Data processing | NA |
| Data | Transfer data | Quantification of Novelty | NA |
| Model | Variable pre-selection | Variable pre-selection | NA |
| Model | Multicollinearity | Multicollinearity | We quantified the correlation levels between abiotic predictors via the Pearson’s correlation coefficient (*r*) and excluded strongly collinear variables (i.e., \|*r*\| > 0.7) to reduce their possible effects on model fitting. |
| Model | Model settings | Model settings (fitting) | GLM: We optimized model parameters via stats R package; GAM: We optimized model parameters via mgcv R package; MaxEnt: We optimized model parameters via ENMeval R package; RF: We optimized model parameters via randomForest R package. |
| Model | Model settings | Model settings (extrapolation) | TRUE |
| Model | Model estimates | Coefficients | NA |
| Model | Model estimates | Parameter uncertainty | NA |
| Model | Model estimates | Variable importance | We estimated permutation importance of marine predictors for Japanese sea cucumber. |
| Model | Model selection - model averaging - ensembles | Model selection | NA |
| Model | Model selection - model averaging - ensembles | Model averaging | NA |
| Model | Model selection - model averaging - ensembles | Model ensembles | Stacked generalization |
| Model | Analysis and Correction of non-independence | Spatial autocorrelation | We corrected for sampling bias in occurrence records by keeping one record per 3 arcmin grid cell. |
| Model | Analysis and Correction of non-independence | Temporal autocorrelation | NA |
| Model | Analysis and Correction of non-independence | Nested data | NA |
| Model | Threshold selection | Threshold selection | We transformed the continuous predictions into presence/absence results using the 10th percentile of the habitat suitability values of species occurrences as binarization threshold. |
| Assessment | Performance statistics | Performance on training data | NA |
| Assessment | Performance statistics | Performance on validation data | AUC; continuous Boyce index |
| Assessment | Performance statistics | Performance on test data | NA |
| Assessment | Plausibility check | Response shapes | NA |
| Assessment | Plausibility check | Expert judgement | NA |
| Prediction | Prediction output | Prediction unit | Habitat suitability, predicted presence/absence |
| Prediction | Prediction output | Post-processing | NA |
| Prediction | Uncertainty quantification | Algorithmic uncertainty | NA |
| Prediction | Uncertainty quantification | Input data uncertainty | NA |
| Prediction | Uncertainty quantification | Parameter uncertainty | NA |
| Prediction | Uncertainty quantification | Scenario uncertainty | NA |
| Prediction | Uncertainty quantification | Novel environments | NA |
